# Delivering integrated hypertension care at private health facilities in urban Pakistan: a process evaluation

**DOI:** 10.3399/bjgpopen18X101613

**Published:** 2018-11-28

**Authors:** Muhammad Amir Khan, John D Walley, Nida Khan, Muhammad Ahmar Khan, Saima Ali, Rebecca King, Shaheer Ellahi Khan, Faisal Imtiaz Sheikh, Farooq Manzoor, Haroon Jehangir Khan

**Affiliations:** 1 Chief Coordinating Professional, Association for Social Development, Islamabad, Pakistan; 2 Professor of International Public Health, Nuffield Centre for International Health and Development, Leeds Institute of Health Sciences, University of Leeds, Leeds, UK; 3 Project Coordinator, Association for Social Development, Islamabad, Pakistan; 4 Research Coordinator, Association for Social Development, Islamabad, Pakistan; 5 Research Coordinator, Association for Social Development, Islamabad, Pakistan; 6 Lecturer, Nuffield Centre for International Health and Development, Leeds Institute of Health Sciences, University of Leeds, Leeds, UK; 7 Assistant Professor, Humanities and Social Sciences Department, Bahria University, Islamabad, Pakistan; 8 Research Coordinator, Association for Social Development, Islamabad, Pakistan; 9 Provincial Manager, Non-Communicable Disease Control Program, Lahore, Pakistan; 10 Director, NCD & Mental Health, Directorate General of Health Services, Punjab, Pakistan

**Keywords:** Integrated care package, private clinics, mixed methods research, hypertension, primary health care, general practice

## Abstract

**Background:**

In Pakistan about 18% of all adults are affected by hypertension, and only one in eight of the prevalent cases have their hypertension controlled. As in many other low-middle income countries, a public–private partnership approach is being considered for delivering non-communicable disease care in urban areas.

**Aim:**

This process evaluation was undertaken to understand how an integrated care intervention was experienced by the care providers and patients, and to inform modifications before possible scaling.

**Design & setting:**

The mixed-methods study was conducted as part of a cluster randomised trial on integrated hypertension care at 26 private clinics.

**Method:**

The care practices were assessed by analysing the clinical records of 1138 registered patients with hypertension. Then semi-structured interviews with service providers and patients were used to understand their respective care experiences. A framework approach was applied to analyse and interpret the qualitative data.

**Results:**

District-led objective selection and context-sensitive staff training helped to get the clinics engaged in partnership working. About one-third of patients with hypertension had associated diabetes or renal compromise. The prescription of drugs is influenced by multiple non-clinical considerations of providers and patients. Many doctors allowed the use of home-based remedies as supplements to the prescribed allopathic drugs. Female patients faced more challenges in managing lifestyle changes. The intervention improved adherence to follow-up visits, but patient attrition remained a challenge.

**Conclusion:**

The integrated hypertension care intervention at private clinics is feasible, and leads to improved diagnosis and treatment in low-income country urban setting. The authors recommend continued implementation research and informed scaling of hypertension care at private clinics.

## How this fits in

Engagement of private clinics in delivering integrated care for hypertension, and other non-communicable conditions, is a known priority but has never been evaluated in Pakistan. The cluster randomised trial and process evaluation studies were conducted to assess the effectiveness and feasibility of delivering integrated hypertension care at private clinics. The process evaluation was undertaken mainly to help to understand and refine the delivery of care tasks in private healthcare settings.

## Introduction

Worldwide, around one billion people are hypertensive (>140/90 mmHg) and this figure is expected to increase by 50% by 2025.^1^ According to National Health Survey Pakistan, about 18% of all adults and 33% of adults aged >45 years are affected by hypertension.^2^ Experience to date suggests that only about half of the people with hypertension are diagnosed; then only half of the diagnosed are ever treated; and then only half of the treated have their hypertension controlled (that is, 12.5% of the prevalent cases).^3^ Performance is higher in developed countries (for example, 24% and 35% hypertension control rate in Australia and US respectively) than in the south Asian countries (for example, 8% and 6% hypertension control rates in China and India respectively).^4^ Hypertension is often the earliest and most common manifestation of the ‘metabolic syndrome’; and may coexist with hyperlipidaemia and diabetes. The common risk factors include: a high salt, fat, and sugar diet (which has become more affordable), and limited physical activity. People with hypertension, especially if diagnosed late and/or not controlled, are at high risk of cardiovascular diseases such as stroke and myocardial infarction.

To standardise the diagnosis, treatmentn and education of patients with hypertension, the provincial Non-Communicable Disease and Mental Health Programme (NCD/MH) developed a case management desk guide, training modules for doctors and allied staff, and a patient education tool. These products were based on adaptation of international best-practice guidelines,^5–9^ with operational modalities added as per country health services and social context.

In Pakistan, the limited governmental primary care infrastructure in urban localities means patients often attend either a secondary or tertiary hospital or a private clinic. The private clinics generally are not oriented to identifying chronic diseases and providing continuity of care. In response to the increasing importance given to partnership working, public–private partnership approaches are being considered for urban areas in low-middle income countries.^10–12^ A district-led partnership approach has been developed and tested for delivering communicable disease care in Pakistan.^13^ In view of these experiences and future considerations, the NCD/MH Programme opted for a district-led partnership approach to engage private clinics in delivering programme-endorsed hypertension care. However, the enhanced hypertension care intervention was not implemented as part of any ongoing healthcare intervention at these clinics.

The enhanced care intervention comprised: (a) mapping and selecting priority localities and private clinics in each priority locality; (b) signing partnership agreements and training the clinic staff on the programme-endorsed care; (c) providing case management desk guides and essential material inputs, for example, blood pressure (BP) apparatus and weighing scale; and (d) monitoring performance and supporting quality care.

A cluster randomised trial found the intervention at private clinics to be effective in reducing the mean systolic blood pressure (≤140 mmHg); and the results are in press.^14^ This process evaluation study was conducted to understand how the context, issues of fidelity, and participants’ responses related to the intervention results.

The study covered the experience of implementing hypertension care for patients (*n* = 1138) at 26 intervention and control clinics in three districts.

## Method

The process evaluation employed both quantitative and qualitative methods in sequence.^1^ The quantitative data from patient clinical records were used to understand the fidelity of the diagnosis and treatment protocols. The qualitative interviews were conducted to understand the experiences of the service providers and patients, and reasons for deviations from the care protocols. The research team and the programme staff jointly discussed and identified five sets of task-categories for studying the intervention. These pre-defined care task-categories were engagement of private clinics; patient identification and diagnosis; treatment and record-keeping; lifestyle modification; and patient follow-up and referral Box 1. Then for each task-category, the team selected a set of quantitative and qualitative indicators. Box 2 shows the logic model for the intervention.

**Box 1.** Selected care tasks and key indicators

Care task	Key ﻿indicators	
	Quantitative	Qualitative
Private clinic engagement	1. Number and/or percentage joined or attended training or withdrew from partnership 2. Patient caseload at clinics	District: selects and engages clinics Clinics: treat a lifelong condition cope: adjusting consultation or drug fees, or operations for dispensing free of cost drugs (provided though the project)
Patient identification and examination for diagnosis	3. Number and/or percentage examined for baseline clinical and laboratory; also age and/or anthropometric measures	Patient’s experiences and provider’s experiences (also practice deviations and reasons) for: identifying and examining overweight and hypertension symptomatic patients conducting clinical and laboratory examinations; and diagnose
Treatment	4. Number and/or percentage of prescribed as per programme guidelines: without comorbid condition with comorbid condition (such as diabetes, renal insufficiency, pregnancy) 5. Number and/or percentage of uncomplicated patient prescribed drugs without trying a lifestyle change6. Number and/or percentage of preventive treatment	Patient’s experiences and provider’s experiences (also practice deviations and reasons) with regards to: prescribing (as per guide) lifestyle trial before drugs prescribing preventive drugs
Lifestyle modification	7. Recording of smoking status (and staff response)	Patient’s experiences of and provider’s experiences (also practice deviation and reasons) for: patient counselling (with pictorial tool) for lifestyle change and smoking cessation
Patient follow-up and adherence	8. Number and/or percentage adhere to follow-up visits (in first 9 months) 9. Number and/or percentage get examined (clinical/ laboratory) on follow-up visits 10. Number and/or percentage referred for expert check-up and/or complication and/or severe drug reaction	Patient’s and provider’s experiences (also practice deviations and reasons) for: patient adherence to follow-up visits (include retrieval) staff adhere to care during follow-up visit referrals (for example,side effects)

**Box 2.** Logic model for the intervention

Intervention inputs	Intervention process and actions	Intended		
		Practice change	Outputs	Health outcome
Case management desk guide and lifestyle counselling tool Training of doctors and allied staff (on full care package) Supplement drugs, equipment and supplies (digital BP apparatus, (glucometer and strips)^a^ Recording forms^a^	Screen/ diagnose^a^ Prescribe antihypertensive Identify comorbid condition and treat Counsel for lifestyle modification Follow-up care, including retrieval	Providers practice programme protocols to: Screen, diagnose, treat, counsel, follow-up and report as per programme protocol Patients practice: Follow-up visits Treatment Lifestyle changes (as counselled)	Patients get: Screened and diagnosed as per programme protocol Prescribed right drug and/or dose Counselled for lifestyle change Followed-up and treated for continued care	Reduction (≥10 mmHg) in mean systolic blood pressure

^a^Inputs and/or practices kept same in intervention and control arms.

The localities in the three districts where private clinics were selected represent poor urban populations with relatively limited access to government facility services. Initially the clinics, in these poor localities, were surveyed for their clinical services and interest in getting engaged in public–private partnerships. All 26 participating private clinics used programme-suggested chronic disease cards for recording the clinical care data on each registered patient. The quality of data was ensured by providing additional training to providers, monitoring the data completeness, and querying any data that appeared implausible. The patient records, collected from October 2014–August 2016, were entered into SPSS (version 17.0). Data were single-entered and the quality was assured by data-entry checking at regular intervals to minimise error rates.^15^ The research team analysed the frequency distribution of the data and cross-tabulated it by sex, and by intervention and control. A preliminary analysis of the quantitative data, performed in February 2016, was also used to inform the design and contents of semi-structured interviews with care providers and patients.

The datasets generated and analysed during the study are not publicly available due to confidentiality of the patient's or responder's identity, but are available from the corresponding author on reasonable request.

### Facility and participant selection

**Figure 1.**
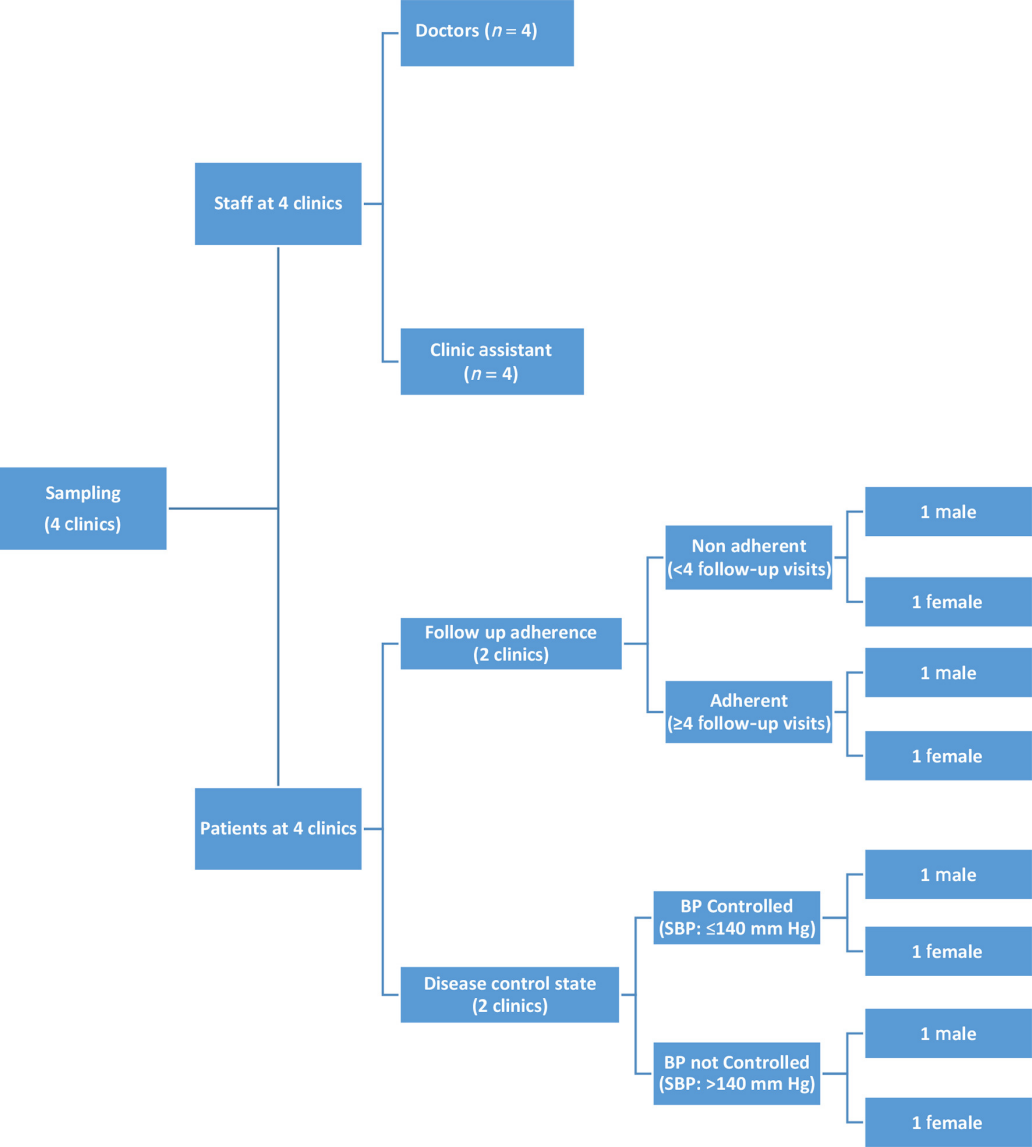
Sampling for staff and patient interviews. SBP = systolic blood pressure.

At four randomly selected clinics, eight designated staff members (that is, one doctor and allied staff at each clinic) and eight purposively selected patients (one male and one female at each clinic) were interviewed. As shown in Figure 1, these patients were sampled according to adherence to appointments (≥4 visits out of the 8 monthly follow-up visits after registration was considered adherent and <4 visits as non-adherent) and control or non-control of systolic BP (>140 mmHg was considered uncontrolled) at 9 months. In April 2016, the patients fulfilling these criteria were identified and randomly selected. Through facilitation of health facility staff (that is, invitation and reminder), all selected patients agreed to take part in interviews at the respective clinic. The consent taken for interviewing a person included their consent to publish any of their interview content without disclosing their identity.

A female researcher, using Skype technology, interviewed the providers and patients in the national language (Urdu) between April–July 2016. After consent, each interview was audio-recorded and lasted for about 30 minutes, with an acceptable level of privacy and confidentiality. A few of the responders were re-contacted by telephone in order to elaborate on their comments and responses if they were not adequately understood.

The researcher who collected the data also transcribed it in Urdu. Then another researcher checked the transcripts, while listening to the recording. The transcripts were anonymised and the identification keys were kept separately from the data. Then these two members of the team, by reading the transcripts, identified the initial codes. A thematic framework was developed, which was based on the five a priori intervention components, and emergent issues were identified jointly by the programme and the research staff, and applied to the data.^15,16^ The indexed data were translated into English, and the quality of translation was checked by other members of the research team. The data from different sources (for example, providers and clients) were triangulated and the key findings and quotations were checked against the original transcripts to ensure trustworthiness and consistency. Ongoing discussion and agreement by the research team framed the analysis process (further information available from the authors on request).

## Results

### Engaging private clinics

Investing a few days of field staff time in the clinic mapping and surveying activity was found feasible and useful. Staff at most private clinics cooperated; that is, they shared the information because of the district health office introductory letter. The score-based prioritisation of surveyed clinics helped making the selection of private clinics credible and acceptable:

*'An introduction letter (from the district health office) helped to make the private clinic staff confident about the surveying exercise.*' (Field staff, Kasur)

About 90% of the invited staff from selected clinics attended the training. No incentive, other than refreshment and travel reimbursement, was offered to the participants. The main factors that seem to have contributed to their attendance included: (a) planning a 2-hour session, which generally is the maximum time clinic staff can afford in a day; (b) keeping the timing of the training session sensitive to their working hours (that is, before or after their clinic hours in the early morning or late evening); (c) selecting, in consultation with the district health office, a neutral venue for the event; (d) sending a formal invitation via the district health office and a reminder through the project staff. The two additional factors that helped to make the training attractive were: (e) imparting skills to deliver care, using programme-endorsed materials and tools; and (f) inviting trainers from the provincial programme and/or institutions. One 'paramedic' (that is, a qualified technical staff member with training in general medical care provision) commented:

*'I found the pictorial tool and skill-learning relevant for our counselling the patients about healthy lifestyle.'* (Paramedic, Clinic District Kasur)

The mean number of patients with hypertension diagnosed and registered at a clinic was around 6 per month (range 1–20). Although the level of contribution varied, none of the engaged clinics withdrew from the partnership.

In general, private clinic arrangements were found more compatible with the care of curable conditions. The clinics seem to prefer taking credit for early cure; not keeping records; and charging a lump-sum fee for consultation and drug supplies for a few days. The reasons for their reluctance to maintain patient records include resource implication (for example, staff time) and administrative considerations (such as tax and legal).

Many clinics reported adapting their arrangements for the continued care of chronic conditions. The adapted care arrangements included: advising patients to visit every month; maintaining patient records; and charging fees for consultation and writing prescriptions (without dispensing drugs). The clinic staff also reported their perceived risk of losing patients if the interval between the two consecutive visits is ≥1 month.

### Examining for diagnosis

Staff interviews suggest that generally the clinics persisted their usual clinical practice; that is, checking BP when a suggestive complaint was reported (for example, headache or blurred vision). The review of 1138 clinical records showed that all patients had their BP checked and labelled correctly (raised systolic [>140 mmHg] and diastolic [>90 mmHg] readings).

All registered patients with hypertension were also assessed for coexisting diabetes and renal conditions. Overall, 355 (31%) of the 1138 newly diagnosed patients with hypertension were found to have raised blood glucose (≥200 mg/dL). This proportion of patients with hypertension with raised blood glucose was found to be similar for male and female patients (30% versus 32%) and at intervention and control clinics (31% versus 31%). Similarly, 820 patients with hypertension (72%) were tested for urine protein, and traces of protein and proteinuria (≥1+) were found respectively in 205 (18%) and 53 (6.5%) patients. Owing to a lack of toilet facilities at the clinics, most patients were required to make a second visit, bringing their urine sample (collected elsewhere) to get tested for urine protein. However, the staff found the dipstick to be feasible for testing urine protein at private clinics.

*'Offering toilet facility at our clinic is not feasible for various cultural and logistical reasons.'* (Doctor, Clinic Mandi-Bahuddin)

A total of 458 (40%) patients with hypertension were found to have raised cholesterol (that is, ≥ 200 mg/dL); with similar rates for male and female patients (43%; 38%). No patient reported any challenge in his or her availing the free-of-cost cholesterol testing, arranged for the trial purposes, at a designated quality-assured laboratory (with a collection centre in each district).

*'Free cholesterol testing at a reputable local laboratory helped achieving higher patient compliance.'* (Doctor, Clinic Mandi Baha-ud-Din)

None of the 580 female patients were reported to be pregnant at the time of registration or during the 9-month treatment follow-up. It could possibly be owing to the clinic staff not assessing or recording the pregnancy.

### Prescribing and keeping records

All patients received antihypertension medication prescribed at the time of diagnosis, that is, without trying lifestyle modification before prescribing drugs. The prescription of drugs at the time of diagnosis seems to reflect both patient expectation and provider routine.

*'It is difficult to satisfy a newly diagnosed patient without prescribing medicine; there is always a risk of losing dissatisfied clients.'* (Doctor, Clinic District Kasur)

Table 1 shows the initial prescription given to patients with and without a known raised blood glucose and/or proteinuria. Out of 756 patients without raised blood glucose and proteinuria: 199 (26%) patients were started with the proposed step-1 regimen (38% in intervention and 15% in control arm); 466 (62%) were started directly (possibly unnecessarily) with the step-2 drugs (56% in intervention and 67% in control arm); and 91 (12%) were prescribed drugs other than the step-1 and step-2 drugs (6.5% in intervention and 18% in control).

**Table 1.** Prescription of antihypertension medication

Arm	No known comorbid diabetes or proteinuria (*n* = 756)						Known comorbid diabetes and/or proteinuria (*n* = 382)			
	Thiazide		ACEi/CCB		Others		ACEi/ARB		Others	
	*n*	%	*n*	%	*n*	%	*n*	%	*n*	%
Intervention	143	37.5	214	56.0	25	6.5	101	52.6	91	47.4
Control	56	15.0	252	67.4	66	17.6	92	48.4	98	51.6
Total	199	26.3	466	61.6	91	12.1	193	50.5	189	49.5

ACEi = acetyl cholinestrase inhibitors. ARB = acetyl cholinestrase receptor blockers.CCB = calcium channel blockers.

The prescription of diabetes drugs was found missing for 141 (40%) patients with raised blood glucose. The staff revealed that at the time of registration, many patients were already taking anti-diabetes drugs, which they continued taking; therefore, the staff didn’t think to record in the respective clinic prescription. About two-thirds (147/214) of the patients with diabetic-hypertension, who were prescribed at the clinic, were recommended the step-1 diabetes drug, that is, metformin.

The intervention seems to have promoted step-wise prescribing of drugs for hypertension and associated raised blood glucose, that is, starting with step-1 drugs rather than the step-2 drugs, which is generally recommended for patients who do not respond sufficiently to the step-1 drugs. It also discouraged the prescription of drugs other than the programme recommendation.

The private doctor’s prescription of drugs seems to be a blend of doctor and patient preferences. The doctor preference seems to be guided by three key considerations: medical need; perceived quality of drug; and perceived affordability to the patient. The patient preference seems influenced by the cost of drugs and its perceived symptom control:

*'Some doctors, getting influenced by fancy packaging and active marketing, do prefer high cost (not necessarily better) pharmaceutical products.*' (Doctor, Clinic District Kasur)

Table 2 shows that about one-third of the patients with hypertension were found eligible to receive preventive medication due to an associated raised blood glucose and/or proteinuria. A significant proportion of eligible patients (that is, about 40% in the intervention group, and >75% in the control group) were found to have not been prescribed the preventive medication, as per the programme protocol. However, the practice of prescribing preventive medication to eligible patients was found to be relatively better in the intervention arm (59.4%) compared with the control arm (22.6%). The staff interviews indicated a need to further simplify the decisionmaking for prescribing the preventive medication:

**Table 2.** Prescription of preventive medication among patients with comorbid diabetes and/or hypertension

Arm	Eligible (comorbid diabetes and/or proteinuria)		Treated	
	*n*	**%** of total	*n*	**%** of eligible
Intervention (*n* = 574)	192	33.4	114	59.4
Control (*n* = 564)	190	33.7	43	22.6
Total (*n* = 1138)	382	33.6	157	41

*'A simplified protocol can possibly enhance the prescription of preventive medication at private clinics.'* (Doctor, Clinic District Kasur)

Most private clinics maintained the clinical records, with the assistance of research field staff, of patients with hypertension included in the trial. Patient interviews indicated that staff updating the records on each follow-up visit potentially adds to patient satisfaction.

*'I do appreciate seeing the clinic staff maintaining my card; and the doctor using the card to advise* [and record] *further treatment.'* (Female patient, Clinic District Mandi-Bahauddin)

### Promoting lifestyle modification

The clinic staff mentioned the challenge of explaining to patients the need for life-long treatment without getting cured:

*'I found it difficult to convince patients to continue taking treatment for the rest of their life, without getting cured*.' (Doctor, Clinic District Mandi-Bahauddin)

In the hope of finding a cure, patients seem to continue exploring alternate sources and/or products; for example, faith-based or traditional medicine, or herbs and natural products. In many cases, these products are used to supplement rather than substitute the prescribed drugs:

*'In addition to drugs, I take two garlic cloves in the morning to help controlling my blood pressure*.' *(Female patient, Clinic District Mandi-Bahauddin)*

The responses of clinic staff varied; a few opposed these practices, whereas most allowed the use of home-based remedies if known not to be harmful and were taken as a supplement (and not as a substitute) to the prescribed allopathic drugs:

*'Patients do use culture-based remedies to supplement the allopathic drugs, which we do not discourage unless deemed harmful.'* (Doctor, Clinic District Kasur)

*'I found it difficult to convince patients not to explore other sources of help to get cured.'* (Doctor, Clinic District Mandi-Bahauddin)

At many clinics, both staff and clients considered the settings to be less than optimal for providing adequate privacy during lifestyle counselling. The duration of lifestyle counselling by the clinic assistant at the time of registration was generally <10 minutes, and for subsequent sessions <5 minutes. The staff and patients did not report any significant logistic or social constraint to patient counselling, except time pressure for the clinic assistant:

*'To avoid longer patient queues, we try to match our pace with the doctor’s consultation.'* (Paramedic, Clinic District Mandi-Bahauddin)

Using the change in weight as a proxy indicator of patient lifestyle modification (that is, healthy diet and exercise), the available data on 129 intervention patients indicates that during the first 6 months of treatment: about one-quarter (23%) of patients reduced their body weight; about half of patients maintained their body weight; and about one-quarter (27%) showed increase in their body weight. The reduced weight can (with caution) be considered to indicate some response to lifestyle modification counselling.

Patients did report challenges to practising the advised daily walk (of half an hour) and dietary changes. The patients reported the following walking difficulties: lack of suitable walking places within and outside their houses; not able to adjust to their daily routines; and non-cooperation of family members. The change of dietary practices (that is, change in food selection and recipes) was found more difficult for patients living in a joint family. This may have resulted from having multiple adults with varied tastes and preferences sharing the same food. Also, most patients found it easier to practise the dietary changes that required their individual action rather than a collective family decision:

*'I was able to stop taking cold drinks and ice cream, even when other members of my family continued using these items.'* (Patient, Clinic District Kasur)

At the time of registration, around one-quarter (27%) of male patients and about 2% of female patients reported that they smoked. Nine months after registration, about one-third of those who smoked reported having stopped smoking in both the intervention (30%) and control (27%) arms.

### Follow-up and adherence

The record review of 3728 patient follow-up visits in the intervention and control arms showed staff compliance of 100% and 85% to checking patients’ BP and weight respectively during the follow-up visits.

**Figure 2.**
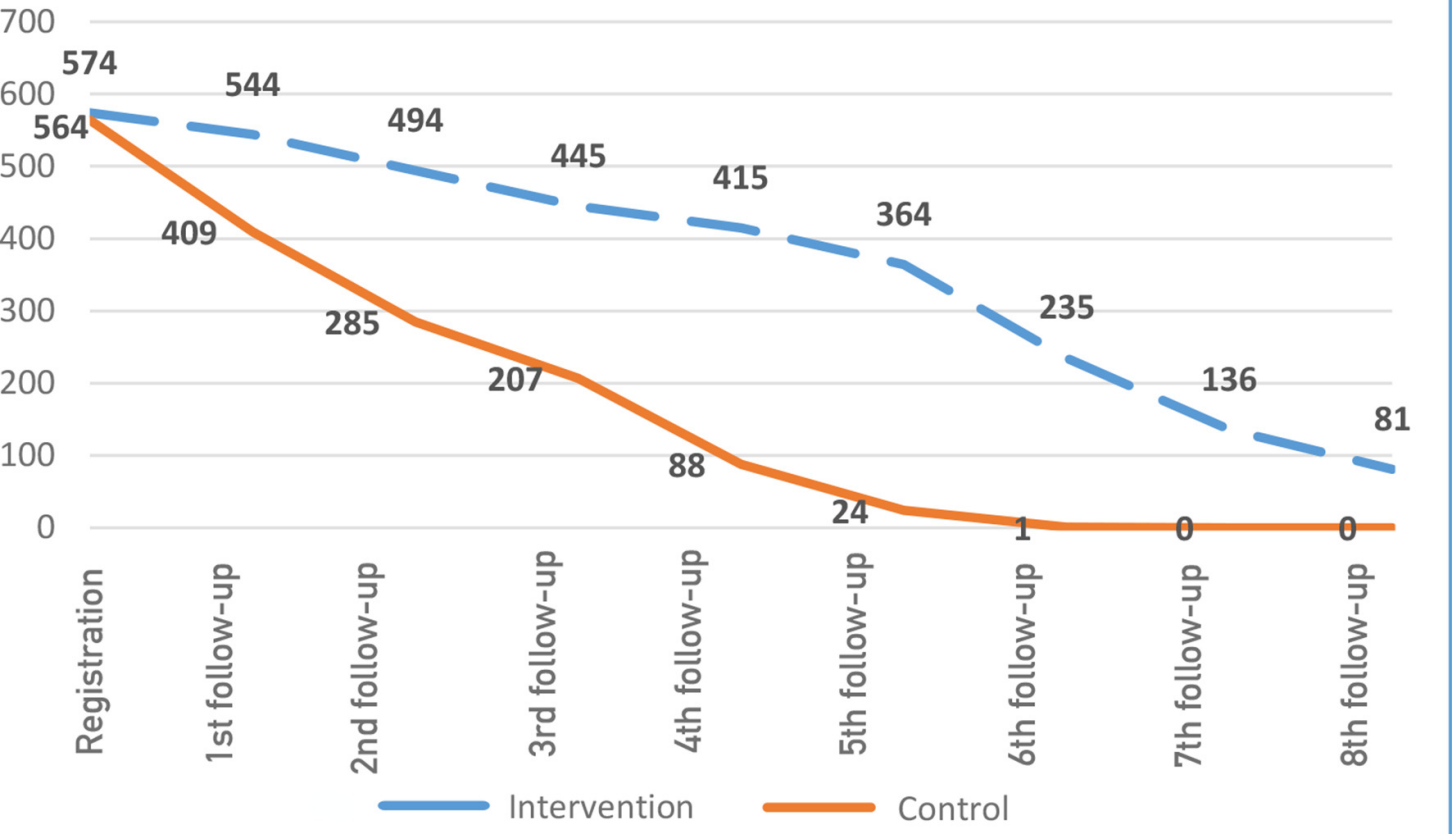
Patient attrition in intervention and control arms.

The compliance to the first 8-monthly follow-up visit requirement was 59% (*n* = 2714/4592) and 22% (*n* = 1014/4512) in the intervention and control arms respectively. As shown in Figure 2, the patient attrition was lesser and slower among those exposed to the intervention (compared with the control). However, patient non-adherence to follow-up visits during the first 9 months seems to have remained a major treatment challenge in both intervention and control arms.

Many patients with hypertension (as a proxy for chronic disease) do not see any added value of monthly visits to the private clinic:

*'I do take the prescribed drugs, but do not see a point in getting myself checked every month.'* (Patient, Clinic District Mandi-Bahauddin)

The intervention sites started a three-tray system, whereby patient treatment cards are moved to the next tray when they attended; while those not attending remain in the first tray and so need a phone recall contact. The relatively better adherence to follow-up visits in the intervention arm indicates the value of this patient retrieval system. However, allied staff reported time and other constraints for their managing the delayed follow-up visits:

*'Patient load makes it challenging* [for a clinic assistant] *to identify and retrieve patients with delayed follow-up visits.'* (Paramedic, Clinic District Kasur)

*'The client can possibly misinterpret our motive* [as commercial rather than clinical] *of reminding him/her about the follow-up visit.'* (Doctor, Clinic District Kasur)

During the first 9 months of treatment, 57 patients (5%) were referred and visited the secondary level facility at least once for a severe health condition. These referral visits were evenly distributed between public and private hospitals; and half of the referral visits (*n* = 28) were for cardiovascular disease-related conditions. The patient and their family decides which hospital to attend in view of the following three key considerations: perceived access; expected quality; and anticipated cost of care. Thirty of the referred patients (53%) reported to have been admitted, for one or more nights, at either a public or private hospital.

## Discussion

### Summary

The cluster randomised trial has shown the integrated hypertension care intervention, with both treatment and lifestyle modification components, at private clinics to be effective in reducing the mean systolic blood pressure (<140 mmHg). The process evaluation study showed that the delivery of integrated hypertension care, as per programme protocols, is feasible for private clinic staff and patients in poor urban settings.

### Strengths and limitations

Using routine clinical records, as the main source of quantitative analysis, made the data collection efficient (minimal added cost), but some variables of interest were not available; for example, education level and occupation. There is lack of literature evaluating the use of Skype technology in relation to interviewing health workers and patients in applied health research in areas, such as participant discomfort or technical failure. However, the authors' experiences were broadly positive and use of Skype technology enabled them to use the expert interviewer’s time more efficiently.

### Comparison with existing literature

The intervention was found to have improved the attendance for follow-up visits. However, many patients seem to consider monthly visits to a private clinic rather unnecessary. A follow-up visit to a private clinic implies paying an access cost and clinic fees; and getting no free drugs from the clinic. The perceived value of paying for a monthly clinic, versus other family responsibilities, may make many patients less certain about using their limited financial resources on clinic attendance (Khan SE, unpublished data, 2018).

The high attrition rate (even in intervention arm) does indicate the need for making treatment adherence easier for patients. Approaches may include providing subsidised or free consultation and/or drugs; and reducing the frequency of follow-up visits (unless clinically indicated).^5^ However, further implementation research is required to learn about providing subsidised care and/or reducing the frequency of follow-up appointments without compromising the clinical requirements; for example, adjusting dosage and/or adding drugs.

This study showed that using a dipstick for testing proteinuria is relatively feasible for private healthcare settings. This seems in line with experiences elsewhere; for example, Patel *et al* ^17^ stated that urine dipstick is feasible and reduces staff workload in routine primary care. Given that about 25% of patients with hypertension had some degree of proteinuria, and the degree of proteinuria does affect the prescription, the authors recommend universal urine dipstick testing of patients with hypertension. In the present study, more than 20% of the female patients were aged ≤35 years, and none were found or reported to be pregnant. The prescription differs if the patient gets pregnant; therefore, point-of-care pregnancy testing should be considered before treating patients who are physiologically capable of pregnancy.

The prescribing practices also improved in line with the programme case management guidelines. However, prescription variation, by ≥﻿20% of the intervention doctors, indicates that further measures need to be explored to achieve universal adherence to the prescription protocols. The literature shows evidence of doctors: (a) advocating specific brands of medicine in response to promotional campaigns of the pharmaceutical companies;^18–20^ and (b) prescribing drugs in response to patient preferences, such as drug cost and perceived quality.^21,22^ In this case, the data were inconclusive as to why some doctors were non-adherent to the programme recommended prescriptions in the case management desk guide.

More recent literature proposes prescription of preventive drugs (statins and low-dose aspirin) if patients are likely to be at high risk; for example, those with diabetes.^23^ The relatively low prescription of these preventive drugs indicates the need to revisit and simplify the prescription protocols. The authors recommend moving towards a criteria-based exclusion approach; that is, excluding aspirin if there is a history of dyspepsia, and excluding statins if significant muscle cramps occur.

Lifestyle change is a complex sociocultural phenomenon that is, mediated by family, community, and gender dynamics.^24^ The study indicated that brief counselling on key messages can be delivered by clinic assistants with the help of simplified (flipchart) tools. The data on response to lifestyle counselling, although inconclusive, indicate difference in responses from male and female patients. Female patients often find it more difficult to do physical exercise and/or change to healthy eating, owing to their social position within the household (Khan SE, unpublished data, 2018). Further qualitative and implementation research is needed to better understand how to promote lifestyle change among patients with hypertension in developing country settings.

### Implications for research and practice

The engagement of private clinics through the district-led partnership approach has been found feasible for delivering care to patients with non-communicable (chronic) disease in a poor urban setting in Pakistan, which is a low-middle income country. However, routine care operations at private clinics do need adjustment for delivering chronic disease (in this case, hypertension) care. In many countries, including Pakistan, a public–private partnership is being considered for expanding the coverage for non-communicable diseases in underserved urban areas. The district-led, public–private partnership seems a potentially feasible and sustainable approach for a scaled implementation of non-communicable disease care at private clinics. However, further implementation research is needed to better understand the scaled implementation in a developing country setting.

This study has shown that integrating hypertension management into routine private clinic care is feasible and acceptable. Integrated hypertension and associated disease care can lead to improved assessment, diagnosis, prescription practices, and adherence to follow-up appointments in a low-income country setting, with promising results. The authors recommend implementation research and other measures for scaled implementation of integrated hypertension care at private clinics in poor urban settings.
